# Changes in the Expression of SNAP-25 Protein in the Brain of Juvenile Rats in Two Models of Autism

**DOI:** 10.1007/s12031-020-01543-6

**Published:** 2020-05-04

**Authors:** Jacek Lenart, Ewelina Bratek, Jerzy W. Lazarewicz, Elzbieta Zieminska

**Affiliations:** Department of Neurochemistry, Mossakowski Medical Research Centre, Polish Academy of Sciences, Pawinskiego 5, 02-106 Warsaw, Poland

**Keywords:** Autism, Rat models, Brain, Gene expression, SNAP-25, RT-qPCR

## Abstract

The results of genetic studies suggest a possible role for SNAP-25 polymorphism in the development of autism spectrum disorders (ASDs); however, there are no data available on whether changes in SNAP-25 expression also affect animals in rodent models of ASD. The aim of the present study was to explore this issue. The studies included 1-month-old rats representing valproic acid (VPA)- and thalidomide (THAL)-induced models of autism. Their mothers received single doses of VPA (800 mg/kg) or THAL (500 mg/kg) per os on the 11th day of gestation. SNAP-25 protein content in the cerebellum, hippocampus, and frontal lobe was determined using Western blotting, while changes of mRNA levels of *Snap25* gene were determined using real-time polymerase chain reaction. Compared to controls, SNAP-25 content was decreased by approximately 35% in all brain structures tested, in both males and females, exclusively in the VPA group. In contrast to this, *Snap25* expression, studied in males, was increased in the hippocampus and cerebellum in both, VPA- and THAL-treated rats. We discuss the compliance of these results with the hypothesized role of SNAP-25 in the pathophysiology of ASD and the adequacy of the experimental models used.

## Introduction

Autism belongs to a group of neurodevelopmental diseases known as autism spectrum disorders (ASDs) (Krzysztofik and Otrebski 2018), which are characterized by impairments in social interactions with restricted and repetitive behaviors and interests (Lai et al. 2014; Stepanova et al. 2017). The role of the genetic component(s) together with epigenetic modifications and environmental factors in the still unknown etiology of ASD has been considered (Bjorklund et al. 2018; Dall'Aglio et al. 2018; Eissa et al. 2018; Lord et al. 2018; Woodbury-Smith and Scherer 2018). Abnormalities in neuronal connections and in neurotransmission play key roles in the pathophysiology of neuropsychiatric disorders, including ASD (Aida et al. 2015; Dickinson et al. 2016; Eissa et al. 2018; Gao and Penzes 2015; Giovedi et al. 2014).

Synaptic transmission is mediated by neurotransmitters released from presynaptic nerve terminals via calcium-dependent exocytosis of synaptic vesicles (Lin and Scheller 2000). The protein complex SNARE (soluble N-ethylmaleimide-sensitive factor attachment protein receptor), which consists of syntaxin, VAMP (vesicle-associated membrane protein), and SNAP-25 (synaptosomal associated protein) plays a crucial role in the mechanism of presynaptic vesicular transport (Hepp and Langley 2001; Rizo 2018). The 25 kDa protein SNAP-25 seems to be attractive candidate proteins for the genetic components of ASD pathogenesis (Cupertino et al. 2016; Ghezzo et al. 2009; Homs et al. 2016; Safari et al. 2017; Wang et al. 2015). Recent genetic studies may suggest a role for SNAP-25 polymorphisms in the development of ASD (Guerini et al. 2011; Safari et al. 2017). Moreover, studies using a SNAP-25 mutant mouse demonstrated anxiety-related behavior (Kataoka et al. 2011), indicating that alterations in SNAP-25 gene structure, expression, and/or function may result in changes in the regulation of emotional behavior and the development of symptoms similar to those observed in neuropsychiatric and neurological disorders (Corradini et al. 2009).

Epidemiological studies in humans demonstrated that exposure to thalidomide (THAL) or valproic acid (VPA) during the first trimester (between the 20th and 24th days of gestation, i.e., at the time of neural tube closure) causes an increased incidence of autism in the offspring (Rodier et al. 1997; Stromland et al. 1994). The corresponding critical period for the exposure of rats to THAL or VPA is on the day 9th to 12th of gestation. The offspring of pregnant female rats treated with THAL or VPA at that time show brain and behavioral abnormalities resembling those found in autistic patients (Ergaz et al. 2016; Narita et al. 2010; Sadamatsu et al. 2006). Based on this data, useful models of chemically induced autism-like behavior in rodents have been developed. In our previous study, using the same VPA and THAL rat models of autism, we also observed behavioral changes similar to autism and neurochemical disruption in the brains of these animals, suggesting disturbances in glutamatergic neurotransmission (Zieminska et al. 2018). We hypothesize that these abnormalities may be a reflection of dysfunction in synaptic vehicle trafficking that result from the altered expression of genes encoding some members of the protein complex SNARE, in particular the SNAP-25 protein and could be implicated in the causation of the behavioral deficits in ASD. However, this hypothesis could not be verified based on the literature because, to our knowledge, there have been no complex studies examining SNAP-25 expression using VPA and THAL rat models of autism.

Thus, the aim of this project was to investigate whether the fetal exposure of rats to VPA or THAL, which are known to induce behavioral deficits similar to ASD and represent the two established animal models of autism, can also modify the expression of SNAP-25 in the brain of juvenile animals. Using Western blotting (WB) and RT-qPCR, we examined the level of SNAP-25 protein, and the expression of *Snap25* gene, respectively, in the cerebellum, hippocampus, and frontal lobe.

## Methods

### Animal Models of Autism

Experiments were performed using male Wistar rats (Cmd: (WI)WU). Rats were bred in the Animal Colony of the Mossakowski Medical Research Centre, Polish Academy of Sciences in Warsaw. The animals were provided water, fed ad libitum, and kept in an air-conditioned room at 20 °C with a constant humidity of approximately 60%, on a 12-h dark-light cycle. All procedures involving animals were in accordance with the Directive 2010/63/EU on the protection of animals used for scientific purposes and with adherence to the national regulations. All of the procedures in animal experiments were approved by the Fourth Local Ethics Committee for Animal Experiments in Warsaw (resolution no. 43/2015 of May 22, 2015).

The procedure of inducing two chemical teratogenic models of autism in rats was performed exactly as previously described (Zieminska et al. 2018). In brief, female rats on the 11th day of gestation were fed by intragastric tube one dose of 800 mg/kg b.w. VPA or 500 mg/kg b.w. THAL. VPA was mixed with 1 ml saline solution, THAL was mixed with vegetable oil, and both were administered orally. Control animals were fed 1 ml of a mixture of oil and saline, 1:1 v/v (Kolozsi et al. 2009; Narita et al. 2010). A random control ultrasonic vocalization test was carried out on PND 9 rats from all experimental and control groups. The results, i.e., a significantly reduced level of ultrasonic vocalization emitted by pups from the VPA- and THAL-treated groups after separation from the mothers, which is considered to be a reliable indicator of pathology similar to autism in rats, did not differ from those described previously (Zieminska et al. 2018).

Newborn rats were bred along with their mothers in individual litters. After 21 days from birth, the pups were separated from their mothers and divided into study groups: control, VPA, and THAL, 3–4 individuals of the same sex per cage. For each test group in our study, the animals came from two litters. At the onset of our experiments, we started with 73 rat pups. Out of the initial number, 1 pup from the control group was excluded from further analysis because of his delay in growth. In the final analysis, there were 24 control animals (9 females—F + 15 male—M), 24 VPA-treated animals (10F + 14 M), and 24 THAL-treated animals (9F + 15 M).

### Western Blotting Analyses

The 35-day-old Wistar rats of both sexes were used for the WB analyses. The animals were sacrificed by decapitation, and the brains were removed from the skull and plated in ice-cold PBS. The frontal lobes (FL), cerebella (CE), and hippocampi (HPC) were isolated from the rat brain, inserted separately into tubes with ice-cold PBS and frozen (− 80 °C) until further analyses. The level of SNAP-25 was determined by the Western Blot performed as described previously (Gamdzyk et al. 2016). Membranes were probed with the anti-SNAP-25 primary antibodies (1:1000; Synaptic Systems GmbH, Göttingen, Germany) and anti-β-actin (1:1000; Sigma-Aldrich) as inner control. Sigma-Aldrich antibodies coupled with alkaline phosphatase were used as secondary antibodies (1:1000). The results are expressed in arbitrary units (arb.u.) as mean ± SD. Statistical analysis of blot data was performed using Kruskal-Wallis ANOVA tests followed by Dunn’s method applying SIGMAPlot 12.5 software package (Systat Software, Inc.). *P* values lower than 0.05 were considered as significant.

### Gene Expression Analyses

For the RT-qPCR gene expression analysis, RNA from three male rats brain region (FL, CE, HPC) was isolated (Total RNA Mini Kit, A & A Biotechnology, Gdynia, Poland), and cleaned (Clean-Up RNA Concentrator kit, A&A Biotechnology, Gdynia, Poland). FL, CE, and HPC brain parts were obtained from the animals based on the method described by Spijker (Spijker 2011).

Expression profiling of the custom gene panel was performed using 10 ng cDNA obtained in RT (iScript advanced reserve transcription supermix (Bio-Rad, Hercules, CA) per reaction. RT-qPCR reactions were performed in 3 replicates by using Sso Advanced Universal SYBR Supermix (Bio-Rad, Hercules, CA) and LightCycler® 96 (Roche Diagnostics GmbH) in the following steps: initial denaturation step at 95 °C for 2 min, followed by 40 cycles of denaturation at 95 °C for 5 s and annealing/elongation at 60 °C for 30 s. The specificity of target amplification was confirmed by melting-curve analysis. RNA integrity was assessed by the 3′/5′ RT-qPCR integrity assay. RNA integrity index was calculated as difference between the 3′Cq (3′ UTR (SDHA3′)) and 5′Cq (5′ end (SDHA5′)), ΔCq = ∣3′Cq-5′Cq∣. RNA with RNA integrity index ΔCq < 1 have been classified as high quality. The primers for this assay were designed and checked by using PrimerQuest Tool (http://eu.idtdna.com) and Primer-BLAST (http://www.ncbi.nlm.nih.gov/tools/primer-blast/) respectively (Table 1). For RNA index quantification, RT reaction was performed by using TranScriba kit (A&A Biotechnology, Gdynia, Poland).

**Table 1:** Primer sequences (and amplicon characteristics) used the 3′/5′ integrity assay

Gene symbol	Gene name	NCBI reference sequence	Primer Sequences (5′–3′) (forward/reverse)	Amplicon length (bp)	Product efficiency E(%)
*SDHA5’*	Succinate dehydrogenase complex flavoprotein subunit A	NM_13042 8	TGGCTTTCACTTCTCTGTTGG	69	2.06
			TGGGTAGAAATCGCGTCTGA		
*SDHA3’*	Succinate dehydrogenase complex flavoprotein subunit A	NM_13042 8	AAGAAGCCATTTGCGGAACA	71	1.89
			GTAACCTTCCCAGTCTTGGT G		

*Actb* (Actin), *B2m* (Beta-2-microglobulin), *Gapdh* (glyceraldehyde-3-phosphate dehydrogenase), *Gusb* (betaglucuronidase), *Hmbs* (Porphobilinogen deaminase), *Hprt1* (hypoxanthine-guanine phosphoribosyltransferase), *Rpl13a* (60S ribosomal protein L13a), *Sdha* (Succinate dehydrogenase [ubiquinone] flavoprotein subunit), *Tbp* (TATA-box-binding protein), *Ppia* (peptidyl-prolyl cis-trans isomerase A), *Ubc* (Polyubiquitin-C precursor), and *Ywhaz* (14–3-3 protein zeta/delta) were chosen as reference housekeeping genes (Augustyniak et al. 2019; Lenart et al. 2017).

The results were analyzed with PrimePCR™ Analysis Software (Bio-Rad, Hercules, CA). Data were presented as fold of changes (FC) of relative normalized expression, assuming that > 4-FC as upregulation and < −4-FC as downregulation (Fig. 2).

## Results

**Fig. 1:**
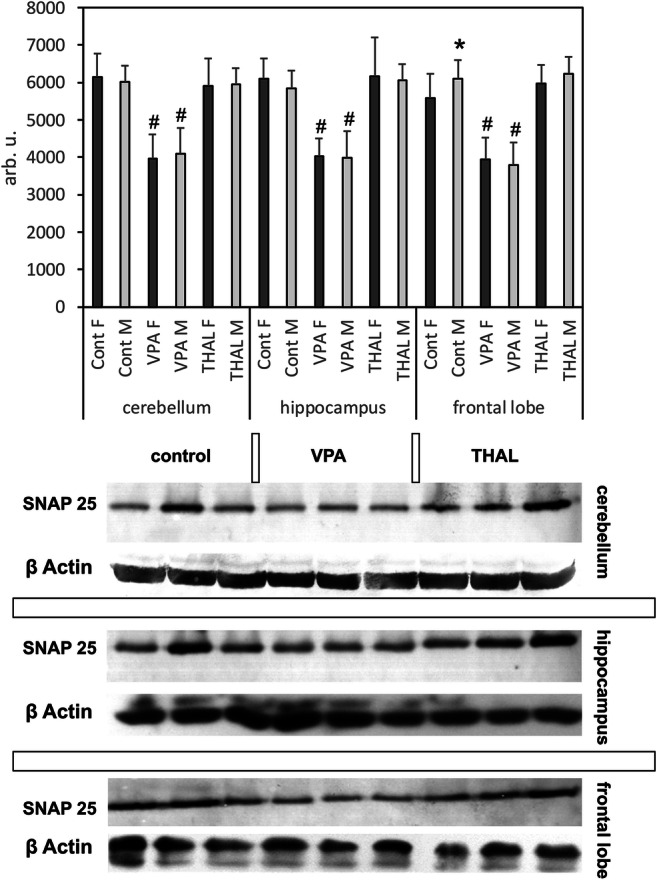
Western blot analysis of SNAP-25 protein content in homogenates of the cerebellum, hippocampus, and frontal lobe of male (M) and female (F) rats in control, VPA- or THAL-treated group. The bar graph shows the densitometric values for SNAP-25, normalized to β-actin, and expressed as arbitrary units (arb.u.). Differences statistically significant vs. * M/F within group or **#** control/VPA, *n* = 24 (9F + 15 M) in control-, *n* = 24 (10F + 14 M) in VPA-, *n* = 24 (9F + 15 M) in THAL-treated group, *p* < 0.05. Below the histograms are presented photos of the full length of blots showing the content of SNAP-25 and β-actin. On these exemplary blots, each group is represented by material from three different male animals

The results of WB analysis examining the SNAP-25 protein levels in the CE, HPC, and FL of rats of both sexes belonging to the VPA and THAL groups are presented in Fig. 1. No significant differences were observed between male and female in analyzed brain structures regardless if we analyzed all groups or each group separately except in FL in control group, where SNAP-25 level was higher in the male group by 9%. There were no changes in the SNAP-25 content in any of the three examined structures of the THAL-treated group. However, in VPA-treated male rats, the SNAP-25 protein level significantly decreased in the CE, HPC and FL compared to the control by 32, 38, and 32%, respectively (Fig. 1). In females from the VPA-treated group, the reduction of SNAP-25 protein levels in the same brain regions was 35, 34, and 29%, respectively.

**Fig. 2:**
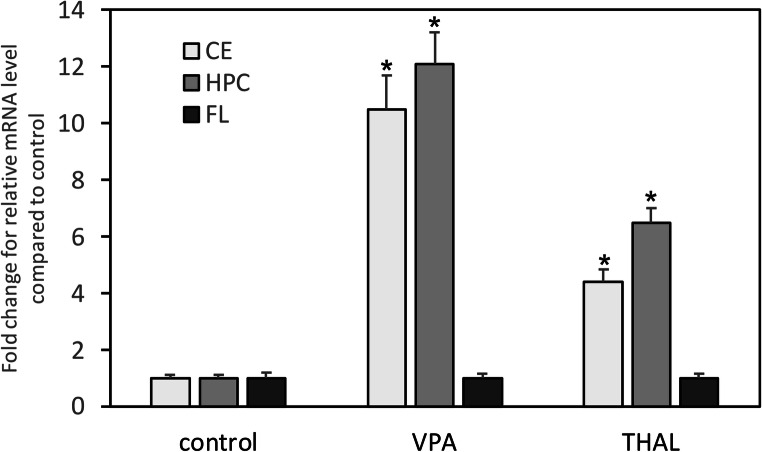
Fold change (mean ± SD) of relative mRNA level compared to control in cerebellum (CE), hippocampus (HPC), and frontal lobe (FL), in control, VPA- and THAL-treated rats. Differences statistically significant vs. * control, *n* = 9 in each group and brain structure *p* < 0.05

After performing RT-qPCR using 96-well plates, the differences in mRNA expression between the control group and rats from the VPA and THAL groups in particular areas of the brain were evaluated. Significant differences in the mRNA levels of *Snap25* between the control and both experimental groups were found in the cerebellum and hippocampus only. In VPA-treated male rats, a 10-fold and 12-fold increase was noticed in the CE and HPC, respectively. In THAL-treated rats, the increase in *Snap-25* expression was lower than in the VPA group; in the former group, a 4-fold and 6-fold increase was found in the CE and HPC, respectively (Fig. 2).

## Discussion

The results of this study revealed differential changes in the level of SNAP-25 protein and the expression of *Snap-25* gene. We noticed a significant reduction in the level of SNAP-25 protein in all three tested structures of the brain only in the group treated with VPA. In both experimental groups, a significant increase in *Snap25* mRNA levels in the hippocampus and cerebellum was observed. These results are consistent with data from human genetic studies indicating the association of proteins involved in synaptic vesicle trafficking, particularly of SNAP-25, with behavioral deficits characteristic for ASD and some other neurodevelopmental diseases. Collectively, these results seem to be consistent with hypotheses suggesting the role of SNAP-25 protein in the pathogenesis of ASD. Moreover, they provide new arguments for the adequacy of the VPA model of autism.

Both, the VPA and THAL groups, represent recognized rat models of chemically induced behavioral disorders similar to autism (Narita et al. 2002; Nicolini and Fahnestock 2018; Schneider and Przewlocki 2005; Schneider et al. 2008; Zieminska et al. 2018). Although repeated epidemiological studies have shown that autism is diagnosed approximately four times more often in boys than in girls (Fombonne 2002), studies on animal models of autism, especially using VPA-exposed animals, have produced varied results, depending on the tests used (Nicolini and Fahnestock 2018). In our previous studies on rats prenatally treated with both VPA and THAL, we also noticed a variable sex dependence of behavioral and neurochemical results (Zieminska et al. 2018). In our current study, we analyzed SNAP-25 protein levels in the experimental and control groups, both in males and females, but we did not find differences between the sexes. Therefore, for ethical reasons, to reduce the number of animals used in experiments, further studies of changes in expression of the *Snap25* gene encoding this protein were performed only in males from VPA and THAL groups. This is in line with the tendency to use only males in most studies on the rodent model of autism induced by VPA (Nicolini and Fahnestock 2018). Although most researchers consider statistically significant at least twofold change in the gene expression, in the present study, using the RT-qPCR method, a fourfold cut-off was used to minimize background noise in the examined animal models of ASD.

This work has focused on the expression of the protein SNAP-25, which belongs to the protein complex SNARE and plays a key role in the mechanism of synaptic vesicle exocytosis and in the modulation of calcium homeostasis in synaptic terminals by regulating the activity of voltage-gated calcium channels (Corradini et al. 2009). SNARE expression changes are suggested to be involved in neurodevelopmental disorders including ASD (Cupertino et al. 2016). More specifically, it has been hypothesized that the *Snap25* polymorphism can be assigned roles in the sex-associated differences in behavioral deficits of patients with ADHD and ASD (Ghezzo et al. 2009). Genetic studies confirmed the association of *Snap25* with ADHD, schizophrenia, and major depressive disorder (Houenou et al. 2017; Wang et al. 2018; Wang et al. 2015); however, apart from the report on the relationship between the *Snap25* polymorphism and ASD in the Iranian population (Safari et al. 2017), the direct association between SNAP-25 and ASD has never been shown in humans. Nevertheless, SNAP-25 is known to play a role in cognitive functions and regulation of locomotor activity, and consequently, associations of this protein with cognitive disorders and hyperactivity have been demonstrated (Guerini et al. 2011; Karmakar et al. 2018; Wang et al. 2018). Other genetic studies investigating autistic children, supported by experiments using the heterozygous *Snap-25*(±) mouse model, have shown a significant association of the *Snap25* polymorphism with reduced cognitive scores in ASD and indicated that the reduced expression of *Snap25* could be responsible for the cognitive deficit (Braida et al. 2015; Braida et al. 2016). Studies using the mouse model with single amino acid mutations in *Snap25* showed the induction of strong anxiety-related behavior (Kataoka et al. 2011). The results of our studies on VPA- and THAL-treated rats showed an approximate 35% decrease in the SNAP-25 protein content in the hippocampus, cerebellum, and in the frontal lobe, exclusively in the former group (Fig. 1). This particular result seems to be consistent with the literature quoted above concerning the pathophysiological role of SNAP-25 (Braida et al. 2015; Houenou et al. 2017; Wang et al. 2018). However, it was surprising to find a very significant increase in the expression of the *Snap25* gene in the hippocampus and cerebellum in both experimental groups (Figs. 1 and 2), whereas based on data from the literature, a reduction in *Snap25* gene expression in ASD animal models could be rather expected.

No positive correlation between mRNA level and encoded protein content is not uncommon, and its mechanisms can be very complex (Liu et al. 2016; Maier et al. 2009). Post-translational modifications of the protein, affecting its rate of degradation, are considered to be the main sources of the absence of correlation between gene expression and protein level (Greenbaum et al. 2003). SNAP-25 undergoes four major post-translational modifications: phosphorylation (Pozzi et al. 2008), palmitoylation (Gonzalo and Linder 1998), S-nitrosylation (Connell et al. 2009), and N-terminal acetylation (Huang et al. 2009). Both, phosphorylation and N-terminal acetylation are currently indicated as most important sources of difference between mRNA and protein contents (Nguyen et al. 2018). Moreover, some types of post-translational modification leading to protein degradation can result from the oxidative stress (Gianazza et al. 2007), and it is worth mentioning that this process occurs in the rat brain in an ASD model induced by VPA exposure (Matsuo et al. 2020). We believe that the mechanism of the difference in *Snap25* expression and this protein level observed in our research can be attributed to some of these phenomena.

The mechanism of increase in *Snap25* gene expression observed in the present study in both ASD models is also unclear. The speculative explanation that this may be a compensatory phenomenon resulting from a decrease in SNAP-25 protein content can only be referred to the group of VPA-treated rats. In a previous in vitro study, we showed that acute exposure of cerebellar granule cells in primary culture to VPA decreased the content of SNAP-25, while THAL induced opposite alteration; moreover, these changes in protein content were consistent with the direction of the mRNA changes (Zieminska et al. 2016). These previous results suggested that SNAP-25 expression may be a target for environmental modification. The changes in *Snap25* expression and SNAP-25 protein levels observed in the current study in vivo could therefore be a direct and persistent result of distant, one-time exposure to both tested teratogens causing the alterations in *Snap25* gene structure, expression, and function. According to an alternative explanation suggested by other authors (Corradini et al. 2009), these may be rather secondary effects of developmental disorders in the central nervous system, caused by the use of teratogens in the critical embryonic period. Further research is needed to determine the exact mechanism of changes in *Snap25* expression and in the level of SNAP-25 protein in the brain of rats treated in the embryonic period with teratogens, particularly with VPA. The same applies to the explanation of the mechanism of differences in the effect of fetal exposure on VPA and THAL on the content of SNAP-25 protein in the rat brain.

## Conclusions

Here, we report differential changes in the level of SNAP-25 protein and in the expression of *Snap25* gene in the brain of juvenile male rats in two chemical models of autism induced by exposure in the critical period of fetal life to teratogens VPA and THAL. This is the first use of rat chemical models of autism to verify the hypothesis that disturbances in SNAP-25 protein expression may be involved in the pathophysiology of ASD. We observed an approximately 35% decrease in this protein content in the hippocampus, cerebellum, and frontal lobe, only in the VPA model, while an increase in the expression of *Snap25*, which encodes the SNAP-25 protein, was found in the hippocampus and cerebellum in both ASD models. These observations are partly consistent with the results of genetic studies investigating children with ASD reported in the literature. This compatibility with previous results was greater in the VPA model. The obtained results are also consistent with the tested working hypothesis and indicate the adequacy of the experimental models used, especially of the VPA model. Further research is needed to explain the mechanisms of the changes in *Snap25* gene expression observed in this work..
